# Are the current notification criteria for Lyme borreliosis in Norway suitable? Results of an evaluation of Lyme borreliosis surveillance in Norway, 1995–2013

**DOI:** 10.1186/s12889-016-3346-9

**Published:** 2016-08-05

**Authors:** Emily MacDonald, Didrik Frimann Vestrheim, Richard A White, Kirstin Konsmo, Heidi Lange, Audun Aase, Karin Nygård, Pawel Stefanoff, Ingeborg Aaberge, Line Vold

**Affiliations:** 1 Department of Infectious Disease Epidemiology, Norwegian Institute of Public Health, P.O. Box 4404, Nydalen, NO-0403 Oslo, Norway; 2 European Programme for Intervention Epidemiology Training (EPIET), European Centre for Disease Prevention and Control, Stockholm, Sweden; 3 Department Bacteriology and Immunology Epidemiology, Norwegian Institute of Public Health, Oslo, Norway; 4 European Programme for Public Health Microbiology Training (EUPHEM), European Centre for Disease Prevention and Control, Stockholm, Sweden; 5 Department of Health Statistics, Norwegian Institute of Public Health, Oslo, Norway

**Keywords:** Public health surveillance, Lyme disease, Borrelia burgdorferi

## Abstract

**Background:**

The approach to surveillance of Lyme borreliosis varies between countries, depending on the purpose of the surveillance system and the notification criteria used, which prevents direct comparison of national data. In Norway, Lyme borreliosis is notifiable to the Surveillance System for Communicable Diseases (MSIS). The current notification criteria include a combination of clinical and laboratory results for borrelia infection (excluding Erythema migrans) but there are indications that these criteria are not followed consistently by clinicians and by laboratories. Therefore, an evaluation of Lyme borreliosis surveillance in Norway was conducted to describe the purpose of the system and to assess the suitability of the current notification criteria in order to identify areas for improvement.

**Methods:**

The CDC Guidelines for Evaluation of Surveillance Systems were used to develop the assessment of the data quality, representativeness and acceptability of MSIS for surveillance of Lyme borreliosis. Data quality was assessed through a review of data from 1996 to 2013 in MSIS and a linkage of MSIS data from 2008 to 2012 with data from the Norwegian Patient Registry (NPR). Representativeness and acceptability were assessed through a survey sent to 23 diagnostic laboratories.

**Results:**

Completeness of key variables for cases reported to MSIS was high, except for geographical location of exposureThe NPR-MSIS linkage identified 1047 cases in both registries, while 363 were only reported to MSIS and 3914 were only recorded in NPR. A higher proportion of cases found in both registries were recorded as neuroborreliosis in MSIS (84.4 %) than those cases found only in MSIS (20.1 %). The trend (average yearly increase or decrease in reported cases) of neuroborreliosis in MSIS was not significantly different from the trend for all other clinical manifestations recorded in MSIS in negative binomial regression (*p* = 0.3). The 16 surveyed laboratories (response proportion 70 %) indicated differences in testing practices and low acceptability of the notification criteria.

**Conclusions:**

Given the challenges associated with diagnosing Lyme borreliosis, the selected notification criteria should be closely linked with the purpose of the surveillance system. Restricting reportable Lyme borreliosis to neuroborreliosis may increase validity, while a more sensitive case definition (potentially including erythema migrans) may better reflect the true burden of disease. We recommend revising the current notification criteria in Norway to ensure that they are unambiguous for clinicians and laboratories.

## Background

Lyme borreliosis (LB), caused by the spirochete *Borrelia burgdorferi sensu lato*, is the most commonly reported tickborne infection in Norway [1]. LB has many different clinical presentations that include dermatological, rheumatologic, cardiac and neurological symptoms [2]. Localized infection is most often characterized by erythema migrans, a rash that often occurs at the site of a tick bite between 3 and 30 days after being bitten. The infection may disseminate weeks to months following exposure and can result in a range of manifestations, including multiple erythema migrans skin lesions, Lyme neuroborreliosis, Lyme arthritis, borrelial lymphocytoma, acrodermatitis chronica atrophicans (ACA) and Lyme carditis [3]. IgM antibodies may be detectable 1 to 2 weeks after tick bite, typically peak during the third to sixth week after onset and demonstrate a gradual decline over several months, while IgG can be detected as early as 2 weeks after onset of disease and can remain at significant levels for many years after onset and after cleared infection [4].

Although diagnosis of the most common clinical presentations of LB is generally straightforward in combination with laboratory results, there is a risk of misdiagnosis for cases with ambiguous clinical presentation or false positive or inconclusive test results. This is particularly relevant in areas with a high prevalence of seropositivity, which can influence pre-test probability of a positive result [2]. The possibility of false positives due to the high testing rate in Norway of up to 60,000 people per year cannot be discounted, despite a high test specificity. Studies have found that 18 % of healthy blood donors from southern Norway [5] and up to 10 % in Western Norway [6] are seropositive for *Borrelia burgdorferi* IgG antibodies. Due to the complexities associated with diagnosis of LB, surveillance of the disease is inherently challenging. The reporting systems and notification criteria used for surveillance in different countries vary and the sources of data are diverse, with physician reports, laboratory reports, hospital records, sentinel surveillance sites, and seroprevalence studies used as sources of surveillance data [7–9]. LB has been notifiable to the Norwegian Surveillance System for Communicable Diseases (MSIS), based on a combination of clinical and laboratory criteria, since 1995. In Norway, the current notification criterium for LB is:

*A clinically compatible case (not erythema migrans only) with laboratory confirmation of Borrelia burgdorferi by:*

- *isolation or nucleic acid test*
- *antibodies (IgM in serum or cerebrospinal fluid (CSF), or IgG in CSF produced intrathecally or with a high concentration in serum).*

*Early localized disease (erythema migrans only) is not notifiable. Multiple erythema migrans is considered disseminated disease and is notifiable.*

**Fig. 1:**
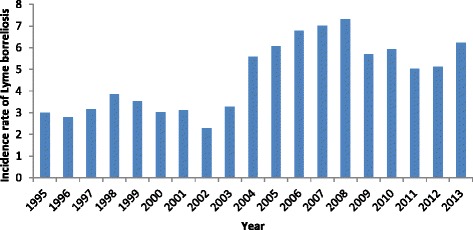
Incidence rate of Lyme borreliosis reported via MSIS per 100 000 population, Norway, 1995–2013

From 1995 to 2013, a total of 4148 cases of LB were reported through MSIS, corresponding to an average annual incidence of 4.7 cases per 100,000 population, ranging from 2.3 in 2002 to 7.3 in 2008 (Fig. 1). However, the incidence varied greatly by geographical area throughout Norway. The vector for *Borrelia burgdorferi*, *Ixodes ricinus,* is found primarily in coastal areas, stretching from the Oslofjord north to Helgelandskysten. However, it is increasingly being found further inland, at more northern latitudes and at higher altitudes [10].

It is suspected that the Norwegian notification criteria are interpreted inconsistently by laboratories and clinicians in Norway. The results of national proficiency testing in 2012 showed that interpretation of diagnostic test results for LB is not uniform among laboratories [11]. Therefore, an evaluation was conducted to assess the performance of MSIS for the surveillance of LB in order to provide recommendations for improvement of the surveillance system. In particular, the results of the evaluation were compiled to assess the suitability of the current notification criteria for surveillance of LB in Norway. Using the Centers for Disease Control and Prevention (CDC) Updated Guidelines for Evaluating Public Health Surveillance Systems [12], the following three attributes of MSIS were selected for evaluation: data quality (which includes completeness), representativeness, and acceptability.

## Methods

### Lyme borreliosis surveillance in Norway

#### Lyme borreliosis in the Norwegian Surveillance System for Communicable Diseases (MSIS)

MSIS was implemented nationwide in 1979 and is administered by the Norwegian Institute of Public Health (NIPH). The stated objective of MSIS is to enable surveillance of infectious diseases in humans in Norway through the ongoing and systematic collection, analysis, interpretation and reporting of data on the incidence of infectious diseases [13]. The data in MSIS is collected in order to 1) describe the incidence of infectious diseases over time, including the geographic distribution and demographic characteristics; 2) detect and contribute to the investigation of outbreaks of infectious diseases; 3) advise the public, healthcare professionals and public health authorities on infection control measures; 4) evaluate the effects of infection control measures; and 5) conduct, promote and provide a basis for research on infectious disease epidemiology and causes of infectious disease. Beyond the general objectives for MSIS, specific objectives for the surveillance of LB in Norway are not defined.

Notifiable diseases in Norway are currently divided into three groups [13]. Group A diseases, which includes LB, are notifiable to the Department of Infectious Disease Surveillance at the NIPH by clinicians and medical microbiological laboratories with complete patient information. Cases of LB were notified sporadically to MSIS from 1983 [1]. Since 1991, LB has been nominally notifiable. In the early years of notification, all manifestations of LB were notifiable, including erythema migrans. The case definition was revised with the implementation of the Infection Disease Control Act in 1995, after which only disseminated and chronic manifestations remained notifiable (specifically excluding cases with only erythema migrans).

#### Diagnosis of Lyme borreliosis

Three types of tests are in use in Norway to detect borrelia: ELISA/chemiluminescence assay and Western blot/line blot for antibody detection, and PCR for detection of genetic material. Line blot testing is available in larger laboratories, but is not required for confirmation of ELISA results. PCR is conducted at three laboratories, including the National Reference Laboratory for borreliosis at Sørlandet Hospital. In Norway it is recommended to use ELISA with VlsE/C6 peptide, rather than the two tired principle recommended in the USA (enzyme immunoassay or immunofluorescence assay, followed by Western Blot). Testing for LB antibodies is most often performed on serum and/or CSF, while PCR for detection of genetic material is done on synovial fluid for cases of Lyme arthritis and on biopsies from skin lesions. Antibody levels are reported as either qualitative values (high, low or borderline value) or as quantitative values, such as a percent of the cut off value or in units defined by the manufacturer of the assay.

#### Data flow

**Fig. 2:**
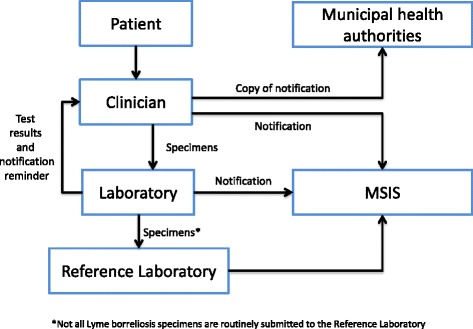
Flow of information from clinicians and laboratories to MSIS

When a patient with a suspected LB seeks healthcare, the clinician sends the relevant specimens to a laboratory (Fig. 2). When a laboratory result indicates LB and submitted clinical information supports notifiable Lyme borreliosis, the laboratory should notify both the requesting clinician and MSIS. For each notifiable case of LB, clinicians are required to submit a standardised paper form to MSIS by mail. The same form is used for all Group A diseases. Clinical symptoms are reported in free text in the notification form. The clinician is also required to send the notification to the Municipal Medical Officer in the patient’s municipality of residence. Laboratory results for LB are not submitted to NIPH using a standard template. When clinical and laboratory notifications are received by the NIPH, the information is manually entered in the MSIS database. The free text clinical information provided by the reporting clinician is used to group reported cases into one of the following categories for clinical presentation in MSIS neurological symptoms with peripheral nervous system manifestation, arthritis, meningitis/encephalitis, necrotizing fasciitis, ACA, other or missing. The notifications from laboratories and clinicians are linked and registered as one case using the personal identification number. The source of the notification (laboratory, clinician or both) is indicated in the MSIS database.

### Evaluation of performance attributes

**Fig. 3:**
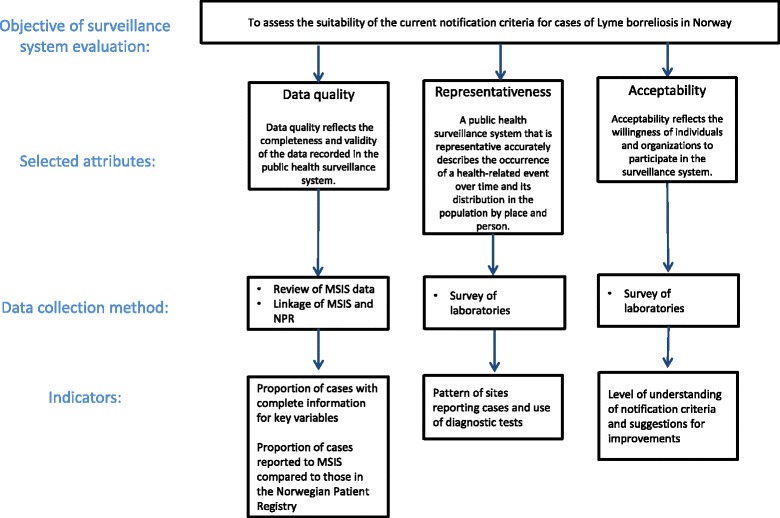
Selected attributes, data collection methods and indicators for evaluation of the surveillance of Lyme borreliosis in the Norwegian Surveillance System for Communicable Diseases (MSIS)

Three attributes were selected for the evaluation: Data quality (including completeness), representativeness and acceptability (Fig. 3). Three methods were used to assess these attributes: 1) descriptive statistics of data in MSIS, 2) linkage between MSIS and NPR, and 3) a survey of laboratories.

#### Data quality

In order to assess the completeness of the data, key variables for all LB records in MSIS from 1995 to 2013 were reviewed to determine the percentage of ‘unknown’ and ‘missing’ responses. The key variables selected were: birthdate; personal number; sex; source of report; reporting laboratory; testing material; testing method, reason for testing; clinical symptoms; hospitalization status; outcome; and geographic location of exposure. Cases are reported as being exposed in their county of residence, exposed outside the county of residence or exposed in an unknown county. All cases with geographic location of exposure reported as unknown or missing, or location of exposure reported as Norway but with the county of exposure either unknown or missing, were categorized as having an ‘unknown county of infection’.

Given the limitations in the available data, it was outside the scope of this evaluation to conduct a conclusive assessment of the validity of the data in MSIS (ie the proportion of cases in MSIS that are true cases of Lyme borreliosis). However, it was possible to compare the registration of Lyme borreliosis cases in two databases to determine the proportion of cases that appear in both sources. In order to ascertain the proportion of cases collected through MSIS that were also registered in the Norwegian Patient Registry (NPR), all cases of LB notified to MSIS between 1 January 2008 and 31 December 2012 were linked to cases of LB registered in the NPR. NPR is administered by the Norwegian Directorate of Health and contains information on all referrals or treatments of patients to tertiary care facilities and specialists only. The diagnosis registered in NPR is based on ICD-10 codes. Codes registered in NPR can be used for differential diagnosis and do not necessarily reflect the final diagnosis. For each entry in NPR, two main diagnoses and 20 additional diagnoses can be recorded. LB is generally coded as A69.2 – Lyme disease (Erythema chronicum migrans due to Borrelia burgdorferi), but can also be coded as M01.2 – Arthritis in Lyme disease. However, coding can vary by hospital and it is possible that other codes can be used, particularly for cases of neuroborreliosis. In order to identify potentially miscoded cases of LB, all patients registered with one of the pre-selected ICD-10 codes between 1 January 2008 and 31 December 2012 were extracted from NPR (Table 1).

**Table 1:** ICD-10 codes pre-selected for possible Lyme borreliosis in the Norwegian Patient Registry

Lyme borreliosis:
• A69.2 – Lyme disease (Erythema chronicum migrans due to Borrelia burgdorferi)
• M01.2 – Arthritis in Lyme disease
Possible Lyme borreliosis:
• G04.2 - Bacterial meningoencephalitis and meningomyelitis, not elsewhere classified
• G04.8 – Other encephalitis, myelitis and encephalomyelitis (Postinfectious encephalitis and encephalomyelitis NOS)
• G51.0 – Bell palsy (facial palsy)
• G51.8 – Other disorders of facial nerve
• G51.9 – Disorder of facial nerve, unspecified
• G63.0 – Polyneuropathy in infectious and parasitic diseases classified elsewhere

Cases of LB in MSIS and patients in NPR with one of the defined ICD-10 codes were linked using personal identification numbers, a unique identifier given to all Norwegian residents, using serial numbers to prevent identification of patients.

Descriptive statistics were produced in order to examine the proportion of neuroborreliosis cases of all LB cases reported to MSIS annually and to identify whether the same pattern was reflected in cases with other clinical manifestations. Cases registered in MSIS with neurological symptoms with peripheral nervous system manifestation or meningitis/encephalitis were considered neuroborreliosis cases, based on the information provided by the reporting clinician in free-text fields. All other clinical manifestations indicated by reporting clinicians were categorized as “other”. The proportion of cases reported to both MSIS and NPR was compared to the proportion of cases reported as neuroborreliosis to MSIS only.

#### Representativeness and acceptability

Representativeness was evaluated through self-administered questionnaires sent to all public and private diagnostic microbiology laboratories in Norway (*n* = 23). Respondents were asked to specify the kit used for diagnosis of borreliosis, how IgG and IgM levels are measured, if thresholds for positive IgG and IgM results are defined, and if standard text is used to report results. The questionnaire was distributed electronically by email using Questback on 13 May 2014. Several questions in the survey distributed to laboratories examined perceptions regarding the current notification criteria and acceptability of the existing surveillance system. Respondents were asked to indicate whether they think the notification criteria for LB are clear, if the diagnostic capacity for LB in Norway is satisfactory, if there is a need for harmonization of diagnostics by using fewer different kits, if there is a need for harmonization of standard text for reporting serology results, and if there was a need for having a kit-independent control.

### Data analysis

Descriptive statistics of data from MSIS and from the laboratory survey were calculated in Excel. Population data at the municipal level used to calculate annual incidence rates were acquired from Statistics Norway. The trend of neuroborreliosis cases versus all other clinical manifestations from 1995 to 2013 and 2004 to 2013 was tested using negative binomial regression. Analysis of the linkage of MSIS and NPR and binomial regression for trend in neuroborreliosis was done in Stata v14.

Ethical approval for the NPR-MSIS study was provided by the Regional Ethics Committee for South East Norway. Written consent was not required from the study population.

## Results

### Evaluation of performance attributes

#### Data quality

Of the 4148 cases of LB reported to MSIS between 1995 and 2013, 85.9 % (*n* = 3 565) were notified by both a clinician and a laboratory. For 161 cases (3.8 %), only clinical notification was received, while for 421 cases (10.2 %) only laboratory notification was received. The proportion of cases reported by both clinicians and laboratories varied with a low of 68.2 % in 2005 to a high of 97.7 % in 2008. With the exception of two cases, all cases reported exclusively by a clinician occurred in 2006 or earlier. From 2006, only cases with a laboratory notification, or notification from both a clinician and the laboratory, were kept in MSIS. Those cases for which only a clinical notification was received were removed from MSIS if no laboratory results were received. It is unknown how many notifications from clinicians only were received after 2006.

Completeness of the key variables selected for surveillance of borreliosis varied from 0 % missing or unknown to more than 40 % missing or unknown. The most complete variables overall (when considering both ‘empty’ and ‘unknown’ as incomplete) were birthdate, personal number, sex and testing laboratory, all with close to 100 % completeness. The variables that were least complete overall were outcome of illness and place of infection, with 43.8 and 35.3 % ‘empty’ or ‘unknown’, respectively. For all variables, the proportion ‘empty’ was larger than the proportion ‘unknown’, with the exception of outcome and geographic location of exposure. When compared to the completeness for all Group A diseases in 2012, LB had a higher level of completeness for all variables with the exception of geographic location of exposure, for which completeness was 89.5 % for LB compared to 91.0 % for all diseases.

The most commonly coded clinical manifestations in MSIS were neurological symptoms with peripheral nervous system manifestation (62.7 %, *n* = 2602), arthritis (12.7 %, *n* = 528 cases), and meningitis/encephalitis (2.2 %, *n* = 93 cases). Necrotizing fasciitis was reported for one case, while 660 cases (15.9 %) were categorized as “other”. Clinical manifestation was unknown for 47 cases (1.1 %) and missing for 216 cases (5.2 %). The proportion of cases with either missing or unknown clinical manifestation ranged from 0 % in 1997 to over 13 % in 2013, almost all due to missing reports from clinicians. Of all cases, 30 % were reported from the National Reference Laboratory for LB, while 36 % of cases of LB that were reported with non-neurological manifestations were reported from the National Reference Laboratory.

**Fig. 4:**
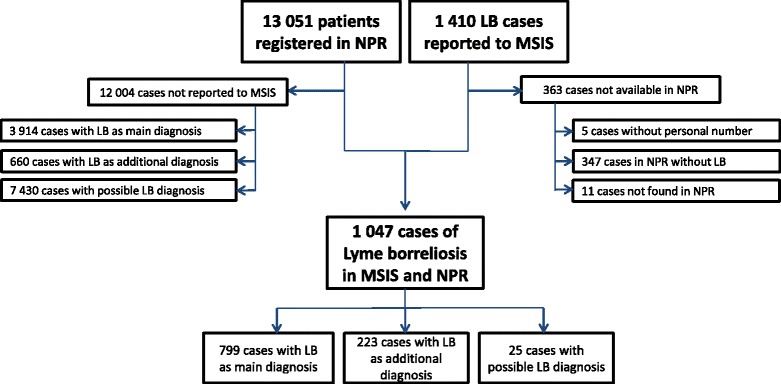
Results of linkage of Lyme borreliosis cases in the Norwegian Surveillance System for Communicable Diseases and the Norwegian Patient Registry

Between 1 January 2008 and 31 December 2012, 1410 cases of LB were reported to MSIS and 13,051 patients were reported to NPR with an ICD-10 code for LB (A69.2 or M01.2) or an ICD-10 that was pre-selected as possible LB (G04.2, G04.8, G51.0, G51.8, G51.9 or G63.0). Of these, 4713 patients had LB recorded as the main diagnosis, 883 patients had LB recorded as an additional diagnosis, and 7430 patients were assigned an ICD-10 code pre-selected for possible LB (but not codes A69.2 or M01.2). A successful link using the personal number could be made for 1047 cases in registered in both MSIS and NPR (Fig. 4). Five cases in MSIS were missing the personal identification number and were excluded from further analysis. Of the remaining 358 cases in MSIS, 347 were found in NPR but did not have any activity reported or were reported with ICD-10 codes other than those included in the linkage. For 11 cases in MSIS, the personal number was not found in NPR. However, these cases were all reported from general practitioners, who do not report to NPR. Over 74 % of LB cases (*n* = 1047) reported to MSIS could be found in NPR with an LB ICD-10 diagnosis code. This constitutes 22 % of patients registered in NPR with LB as the main diagnosis (1047/4713), or 8 % of the patients with any LB ICD-10 code in NPR (1047/13,051). For the 25 LB cases reported to MSIS and registered in NPR with a possible LB diagnosis code, 19 cases were registered with ICD-10 code G51.0 - Bell palsy (facial palsy). Over 38 % of cases registered in both NPR and MSIS were under the age of 19, compared to 15 % in NPR only and 26 % in MSIS only. More than 36 % of cases in MSIS only were over 60 years compared to 25 % in NPR/MSIS and 30 % in NPR only.

Between 1995 and 2013, the proportion of cases recorded as neuroborreliosis in MSIS (neurological symptoms with peripheral nervous system manifestation or meningitis/encephalitis) ranged from 49.6 % in 1995 to 64.4 % in 2006, with an overall average of 64.4 %. Using all relevant available data from 1995 to 2013, the trend (average yearly increase/decrease over this time period) of neuroborreliosis cases versus all other recorded clinical manifestations was not significantly different in negative binomial regression (*p* = 0.261). From visually observing the data, differing trends appeared to occur from 2004, so a post-hoc analysis was undertaken: From 2004 to 2013, the trend of neuroborreliosis cases versus all other clinical manifestations was still not significantly different in negative binomial regression (*p* = 0.136), however, the p-value did numerically decrease while the number of data was reduced. Between 2008 and 2012, more cases reported in MSIS and NPR were registered as neuroborreliosis (84.4 %) than those reported to MSIS only (20.1 %) (Table 2).

**Table 2:** Neuroborreliosis cases (neurological symptoms with peripheral nervous system manifestation or meningitis/encephalitis) by source of data and diagnostic code, 2008-2012

Source	Diagnosis code	Neuroborreliosis	Other clinical	Missing clinical
NPR and MSIS	Main diagnosis (*n* = 799)	609 (76.2 %)	132 (16.5 %)	58 (7.3 %)
	Additional diagnosis (*n* = 223)	186 (83.4 %)	19 (8.5 %)	18 (8.1 %)
	Possible diagnosis (*n* = 25)	24 (96.0 %)	0 (0 %)	1 8 (4.0 %)
MSIS only	Not applicable (*n* = 358)	75 (20.1 %)	243 (67.9 %)	40 (11.2 %)

#### Representativeness

A response to the questionnaire was received from 16 laboratories, of which 15 perform diagnostic testing for borrelia. Two assays for detection of IgM and IgG antibodies were used by 14 (93 %) of the laboratories. However, the practice used for quantification and reporting of Ig-levels differed between units and % of cut-off. The definition of ‘high level IgG’ differed between laboratories using the same diagnostic assay. Only one respondent was positive to limit the number of different assays in use. Seven respondents (44 %) were positive to implementing uniform comments on laboratory reports, and 13 (81 %) wanted a common assay-independent standard serum specimens for calibration and quality assurance purposes.

#### Acceptability

Only 36 % of respondents indicated that the current notification criteria for LB were clear. Half of respondents indicated that their respective laboratory had established internal procedures or algorithms for determining which LB cases should be notified to MSIS, based on laboratory findings and clinical information. Three respondents commented that in practice only neuroborreliosis is notified, while one laboratory would notify all IgG positive specimens to MSIS.

## Discussion

This evaluation of the data quality, representativeness and acceptability of the surveillance of LB through MSIS has identified several strengths and weaknesses of the existing system. Although no objectives have been developed explicitly for the surveillance of LB, perhaps the most important specific objective for MSIS in the context of LB is to describe the incidence of infectious diseases over time, including the geographic distribution and demographic characteristics. Although not perfect, the data currently collected does give an indication of areas of the country where the disease is most prevalent. In addition, because the current notification criteria have been in place since 1995, it is possible to make some conclusions regarding trends over the last twenty years, assuming notification practices have not changed. However, the results also indicate that interpretation of the data collected using the current notification criteria must be made with caution. The major limitation of the current system is that there are evident inconsistencies in what is being reported, particularly from laboratories, indicating that the current notification criteria are either not understandable or intentionally not being followed.

The overall completeness of MSIS data was high, with the exception of geographic location of exposure and outcome of illness. The low completeness of the variable “outcome of illness” was likely due to lack of updated information. There is a long duration of illness for some manifestations of the disease, and updated information from the clinician is seldom available. Reports originating from laboratories may not have the necessary information to report outcome of illness. In contrast, lack of completeness in geographic location of exposure may have the following explanations: Firstly, the patient may not know where they were infected, particularly if they do not have any recollection of being bitten by a tick. Secondly, the clinician may not have asked the patient where they were infected. Thirdly, the clinician did not notify the case to MSIS, resulting in missing clinical information for multiple variables, which has occurred for 10 % of cases. If MSIS information is collected to describe the incidence of infectious diseases over time, including the geographic distribution and demographic characteristics, complete information on place of infection is vital.

There are several findings that suggest that the validity of some information in MSIS, in particular whether all reported cases fulfill the notification criteria, is questionable, although this was not specifically investigated as part of this evaluation. Information on clinical symptoms, which is a part of the notification criteria, is only reported in free text in the physician’s notification form and the coding of clinical presentation is done upon data entry into MSIS. As the free-text information is not collected systematically, it is not possible to determine whether all cases reported meet the current case definition using the free-text information. It is therefore not possible to determine the predictive positive value of notified cases as the determination of whether a case has “clinically suspected disseminated or chronic disease” is the responsibility of the reporting clinician. As 15.9 % (*n* = 660) of cases reported to MSIS are categorized as having a ‘other’ as clinical presentation, this may indicate that many reported cases present with symptoms that are not easily categorized into one of the existing codes. A cursory review of the free-text information suggests that for at least 12 of the 256 cases reported in 2012 the clinical diagnosis of LB was uncertain. Many of these patients have serological test results that are consistent with either a current or previous LB infection but have ambiguous or diffuse symptoms that may not be correctly attributed to LB. In addition, the comparison of data from MSIS and NPR indicates that the cases registered only in MSIS are unlike the cases registered in both MSIS and NPR, both in terms of age and clinical presentation. Almost 80 % of cases in both MSIS and NPR are registered as having neuroborreliosis compared to just over 20 % of those in MSIS only, and are younger than those cases registered in MSIS only.

In addition, there are a number of people who are assigned diagnostic codes for LB in NPR that are ultimately not reported to MSIS, including almost 4000 patients who have A69.2 or M01.2 registered as a main diagnosis. It is not unexpected that there are more patients registered with the ICD-10 code for LB than cases notified through MSIS for several reasons. The ICD-10 codes used in NPR are often assigned to patients while undergoing diagnosis but may not ultimately reflect the final diagnosis. For this reason, patients in our analysis included those who had been assigned one of the ICD-10 codes predefined as possible Lyme borreliosis. These codes were pre-selected in order to identify patients with compatible symptoms that may not have been correctly assigned a Lyme borreliosis-specific ICD-10 code. It was anticipated that in most cases patients in NPR that have been assigned the codes that were selected as possible LB diagnoses are unlikely to be true LB cases. Nevertheless, given that 25 cases registered in MSIS had been registered in NPR with one of these possible LB codes, it cannot be discounted that some of the 7430 cases with possible LB ICD-10 codes in NPR are in fact true cases. The ICD-10 codes for LD also include erythema migrans, which is not reportable to MSIS. However, as NPR registers referrals or treatments of patients to hospitals and specialists only, it is unlikely that many of these cases presented with erythema migrans only. An earlier study linking cases of tuberculosis in NPR and MSIS showed that there are many limitations to how directly these data can be compared, partially due to incorrect assignment of the ICD-10 codes in NPR [14]. However, it is also possible that the significant discrepancy between NPR and MSIS can be explained by significant underreporting of cases that meet the notification criteria. NPR coding also contributes towards hospital reimbursement schemes, which may lead to a greater incentive to register in NPR than in MSIS.

The representativeness of data in MSIS is also limited due to the different testing procedures and reporting practices at laboratories. As the case definition includes laboratory test results, which the clinician must receive before reporting the case, the laboratory plays a vital role for surveillance. It is assumed that most notifications are initiated by the laboratory, as laboratory-based surveillance is the back-bone of the Norwegian notification system. It is difficult to determine how big of an impact differences in laboratory practices and use of different assays have on describing the epidemiology of the disease and on determining the geographical spread. It is possible that in areas where the disease is common, the representativeness is likely to be better as clinicians are more familiar with the disease and are therefore more likely to suspect the diagnosis and ensure appropriate testing. Conversely, in areas where the disease is common clinicians may be more likely to assume LB is the correct diagnosis in situations where the diagnosis is equivocal. Regardless, the low acceptability is likely more challenging to overcome for surveillance than the differences caused by the use of different assays, which can be addressed by harmonizing how results are reported by laboratories. The unclear and different use of diagnostic cut-off values and interpretation of ‘high level IgG’ affect algorithms for notification developed within laboratories whereas the heterogenous reporting of results is problematic for the clinician who has to interpret the results and for the patient. One means of accomplishing harmonization is to develop standard text for reporting and to establish standard cut-off values for antibody levels and indexes. However, there is not consensus on harmonization of thresholds or values for quantification.

Based on this information, it is apparent that there is a need to improve the existing case definition in Norway. However, there is no international consensus on the best approach to LB surveillance and a number of different approaches to LB surveillance are being used throughout Europe. A 2010 survey found that 23 of 28 responding European countries had surveillance systems in place for LB, of which only 16 included mandatory reporting requirements [9]. Some countries, as well as some federal states in Germany, require notification of erythema migrans, as well as neuroborreliosis and Lyme arthritis [15]. In France, a prospective study through a sentinel network of general practitioners used a case definition based on criteria developed by the European Concerted Action on Lyme Borreliosis, which includes either the presence of erythema migrans, or the appearance of neurological, articular, cutaneous or cardiac symptoms evocative of Lyme disease, in a patient with positive serology [16]. Other countries, like Norway, require notification of disseminated infection only. In other countries, such as Denmark, only neuroborreliosis is notifiable (a confirmed case is a patient with clinical symptoms consistent with Lyme neuroborreliosis and a positive antibody index test, while a probable case is a patient with clinical symptoms consistent with Lyme neuroborreliosis and borrelia antibodies in serum) [17]. Although interest has been expressed in developing a common European case definition to allow cross-national surveillance and comparison between countries, the range of surveillance methodologies and laboratory practices across countries has so far made this an unworkable proposition. In addition, there must be consensus on the objectives of surveillance of LB before a common case definition can be introduced. A European expert consultation on surveillance of tick-borne diseases hosted by the European Center for Disease Control concluded that the unknown burden of disease associated with LB is one of the primary incentives for establishment a common case definition [9]. However, due to problems of misdiagnosis and over-diagnosis, the surveillance of LB should aim for specific rather than a sensitive system. Particularly as the disease does not occur in outbreaks, knowledge of each single case is not needed in order to assess general trends. Nonetheless, geographical location is an important aspect of the disease and data needs to be collected at a sub-national level in order to accurately follow trends, particularly in high-risk areas or new foci.

There are several options available to improve the notification criteria for LB in Norway. The first option is to maintain the existing notification criteria, but introduce more specific requirements regarding the laboratory results’ cut-offs and develop strategies for collecting sufficient information to definitively categorize cases by clinical presentation (in order to differentiate neuroborreliosis from other clinical manifestations). In order to reliably distinguish neuroborreliosis cases from all other Lyme borreliosis cases, it will be necessary to modify the current notification criteria to define clear clinical and laboratory criteria for reporting a neuroborreliosis case. Although the categories currently used in MSIS give some indication of the proportion of all cases that are neuroborreliosis cases, the free-text information is currently insufficient to confirm whether a case is “clinically compatible”, a determination that is at the discretion of the reporting clinician. There are several other notifiable diseases that are already reported separately to MSIS depending on the clinical presentation and laboratory diagnosis, such as Hepatitis B (for which acute and chronic infection are reported separately). As Norway still principally uses a paper-based system for notification, adoption of an electronic form that allows for tailored reporting for specific diseases may ease the systematic collection of information on clinical presentation.

The second option is to make the criteria more sensitive, by including erythema migrans. The added value of receiving information on all cases of erythema migrans is questionable, given the burden this would add on reporting clinicians. The third option is to make the criteria more specific, by only requiring notification of neuroborreliosis. Neuroborreliosis diagnosis is considered to improve validity of surveillance data as the clinical picture is more specific and the laboratory methods are more conclusive [9]. As knowledge of each individual case is not needed to assess general trends, a less sensitive but more acceptable system could lead to better data validity. However, using these restrictive notification criteria may produce a species-dependent clinical picture. Some borrelia types are more commonly associated with neurological manifestations (*B. garinii* and *B. burdorferi ss*), while *B. afzelii* is associated with acrodermatitis chronica atrophicans and Borrelia burgdorferi sensu stricto is more often associated with arthritis [18]. All borrelia types are associated with erythema migrans. The implications of only conducting surveillance on one clinical presentation could mask some unknown trends. In addition, burden of disease would be more difficult to ascertain, although this is already limitation of the current notification criteria, in which erythema migrans alone is not notifiable. Given the challenges with the surveillance of LB, there is no gold standard for notification criteria. Any changes to the notification criteria will need to be weighed against the consequences of being unable to compare directly with past data. With the existing weaknesses in the data collected, changes to the notification criteria based on sound evidence could lead to better quality data in the future. The results of this evaluation support that it may be advisable to change the Norwegian notification criteria, particularly as the acceptability among laboratories is low. The current notification criteria include some elements which may lead to ambiguous cases, such as allowing for IgM in serum. The notification criteria must be clear for all users, particularly as the current notification criteria are include both clinical and laboratory elements. It may also be advantageous to ensure that the Norwegian notification criteria are aligned with the EU case definitions [2], in order to ease direct comparison with other countries in the case that LB becomes notifiable in the European region. Ultimately, the determination of the appropriate notification criteria for Norway must be closely linked to the purpose of the surveillance system.

Several studies are currently underway to determine what changes would be most appropriate. A seroprevalence study is being conducted to describe regional difference in antibody prevalence in the general population in order to determine the pretest likelihood of a positive result and to develop a common scale for semi-quantitative reporting of results. A systematic review of patient journals within NPR is also being conducted to determine the validity of ICD-10 codes reported. This will give an indication of how widely cases are underreported and help determine whether cases reported to MSIS fill the notification criteria. Other studies being considered include a kit independent control to see how much the results from different laboratories vary, as well as an investigation of the acceptability of the notification criteria among clinicians, as this information has currently only been collected from laboratories.

## Conclusions

The results of this evaluation suggest that there is a clear need to review the current notification criteria in order to ensure that they are unambiguous for clinicians and laboratories. Given the challenges associated with surveillance of LB, the selected notification criteria should be closely linked with the purpose of the surveillance system. Developing specific objectives for the surveillance of LB may help guide this process. If the primary objective of the system is to describe the incidence of infectious diseases over time, including the geographic distribution and demographic characteristics, restricting reportable LB to neuroborreliosis may increase validity, while a more sensitive case definition (potentially including erythema migrans) may better reflect the true burden of disease. Several key steps for improving surveillance are to identify ways to harmonize notifiable laboratory information and to identify strategies to distinguish between neuroborreliosis and other clinical manifestations. Any benefits associated with changing the notification criteria will need to be weighed against the consequences of limiting the comparability of future surveillance data.

## Abbreviations

ACA, Acrodermatitis chronica atrophicans; CDC, US Centre for Disease Prevention and Control; CSF, Cerebrospinal fluid; LB, Lyme borreliosis; MSIS, Norwegian Surveillance System for Communicable Diseases; NIPH, Norwegian Institute of Public Health; NPR, Norwegian Patient Registry; PCR, Polymerase chain reaction
